# Editorial: Social-related biomarkers and potential noninvasive treatments for sub-clinical and clinical emotional disorders

**DOI:** 10.3389/fpsyt.2023.1254197

**Published:** 2023-08-07

**Authors:** Xiaolei Xu, Luke Sy-Cherng Woon, Lei Xu, Jiaojian Wang

**Affiliations:** ^1^School of Psychology, Shandong Normal University, Jinan, Shandong, China; ^2^Department of Psychiatry, Faculty of Medicine, The National University of Malaysia, Kuala Lumpur, Malaysia; ^3^Institution of Brain and Psychological Sciences, Sichuan Normal University, Chengdu, Sichuan, China; ^4^State Key Laboratory of Primate Biomedical Research, Institute of Primate Translational Medicine, Kunming University of Science and Technology, Kunming, China

**Keywords:** emotional disorders, neuro-biomarker, fMRI, EEG, brain

Emotional disorders, such as major depressive disorder (MDD) and anxiety disorders, are characterized by highly debilitating impairments in emotion regulation and cognitive control which may lead to social dysfunctions. An increasing number of neuroimaging studies have determined several structural and functional alterations in emotional disorders which enhanced our understanding of the pathogenesis of these disorders. Moreover, determining disorder-specific neural abnormalities help to develop more efficient non-invasive interventions. For example, real-time neurofeedback training in the amygdala activity or amygdala-prefrontal connectivity and rTMS targeting the dorsolateral prefrontal cortex have exhibited promising effects in anxiety and depression (1–3). However, there are still approximate one third of patients cannot benefit from the current interventions. Therefore, determining more specific neural biomarkers and taking the age of patients and subtypes of different emotional disorders into account may increase the effects of precise treatment in the future.

This Research Topic included four neuroimaging studies covering patients from adolescence to late-life with either depression or obsessive-compulsive disorders. Sharpley et al. evaluated the association between depressive behavior (DB) subtypes and frontal lobe asymmetry (FLA) using an EEG data network analysis. They found that: (a) the four DB subtypes exhibited significant differences in symptomatology reflected by the Zung Self-rating Depression Scale; (b) the four DB subtypes showed distinct FLA-neurophysiological profiles; (c) the direct and inverse relationships between DB subtypes and FLA data may be the potential confounders leading to inconsistent results of overall FLA-MDD correlations reported in previous studies. This study suggested that the neuropathogenesis may vary in different depression subtypes. Therefore, precise interventions should be subtype-specific or even individual-specific. Cao et al. examined the relationship between insomnia symptoms and suprachiasmatic nucleus (SCN) functional connectivity in depressed adolescents. Their results indicated only patients with high insomnia (MDD-HI) exhibited significantly decreased functional connectivity between right SCN and bilateral precuneus. Thus, altered SCN-precuneus connectivity may represent a potential non-invasive target for depressed adolescents with high insomnia. The study from Shao et al. further identified the differences in gray matter volume (GMV) among late-life depression (LLD) with insomnia, LLD, and healthy controls (HC). They found that the GMV of the anterior lobe of the cerebellum decreased significantly in LLD compared with HC and such abnormality was positively associated with anxiety levels. Their results suggested that the cerebellum may be the potential neural target for non-invasive interventions in LLD patients. The study from Zhang et al. focused on obsessive-compulsive disorder (OCD) and found that OCD patients exhibited significantly higher fractional anisotropy (FA) and lower radial diffusivity (RD) at the level of the insular portion and temporal portion of the left uncinate fasciculus compared with HCs. In addition, increased FA was positively associated with anxiety level, while decreased RD had a negative association with the duration of illness. This study suggested that the focal abnormalities in the uncinate fasciculus may represent the potential neural markers for OCD.

To conclude, the four papers in this Research Topic determined disorder-specific potential neural biomarkers for depressed patients across different age from adolescence, adulthood, and late life, as well as for patients with OCD. While the altered SCN-precuneus connectivity may be a potential neural biomarker for depressed adolescents the anterior lobe of the cerebellum may represent the neural biomarkers for late life depression. Furthermore, the EEG-FLA may be the potential marker to distinguish subtypes of MDD and the uncinate fasciculus may be a potential interventional target for OCD. Future research should continue to determine more specific biomarkers which may contribute to social functioning and examine the effects of non-invasive interventions targeting these biomarkers for different emotional disorders.

## Author contributions

XX: Writing—original draft, Writing—review and editing. LW: Writing—original draft, Writing—review and editing. LX: Writing—original draft, Writing—review and editing. JW: Writing—original draft, Writing—review and editing.
